# Effectiveness of Treating Vertical Maxillary Excess Among Non-growing Adult Patients With Skeletal Anchorage System: A Systematic Review and Meta-Analysis of Case Reports From 2008 to 2023

**DOI:** 10.7759/cureus.76625

**Published:** 2024-12-30

**Authors:** Priti Kiran, Nivedita Sahoo, Bhagabati Dash, Biswaroop Mohanty, Sanghamitra Jena

**Affiliations:** 1 Orthodontics and Dentofacial Orthopaedics, Kalinga Institute of Dental Sciences, Bhubaneswar, IND

**Keywords:** adult, bone screw, buccal shelf, gummy smile, izc, miniplates, miniscrew, sas, tad, vme

## Abstract

Vertical maxillary excess presents a complex challenge in orthodontic treatment, necessitating effective anchorage systems for optimal correction. This research is useful to assess the skeletal anchorage system's (SAS) effectiveness in correcting the vertical maxillary excess among adult patients presenting with gummy smiles. This study includes case reports with English full text and examines the global general adult (18+) human population with vertical maxillary excess (VME). Publications from 2008 to 2023 were considered. The current systematic Review’s framework follows patient, intervention, comparison, outcome and (sometimes) time (PICO(T)) criteria. Information sources were PubMed advanced search, Excerpta Medica database (EMBASE) (Elsevier), Web of Science, and Scopus (advanced search). On February 6th, 2024, the last search was carried out utilizing the same databases; the risk of bias was evaluated utilizing the National Institutes of Health (NIH) Quality Assessment Tool. Data synthesis was carried out on their own by the case and classical teams. The Meta XL (v.5.0) program (EpiGear International Pty Ltd., Queensland, Australia) Excel extension for meta-analysis was used. The inverse variance heterogeneity model was applied during the meta-analysis process. I2 and Cochrane's Q statistics were applied to gauge the degree of data heterogeneity. Two hundred fifty-two potential articles were found as a result of the search technique. Following Preferred Reporting Items for Systematic Reviews and Meta-Analyses (PRISMA) guidelines, a total of 12 studies went through the analysis procedure. All 12 studies (case reports) and a total of 12 participants, including 11 females and one male, were studied from countries like India, the USA, Korea, Japan, China, and Mongolia. The age of patients is between 18 and 36 years. Out of 12 studies, in 11 studies, the orthodontic problem was Class II malocclusion with vertical maxillary excess. The pooled intrusion for the orthodontic treatment was seen, and almost 70% of the studies had a similar range. Only three studies were out of the 95% CI range of the other studies. The heterogeneity among the studies was 97.42%. Findings showed that p was statistically significant (p < 0.0001). The limitation of this study is that this is a systematic review and meta-analysis of case reports.

## Introduction and background

Vertical maxillary excess (VME) is the central skeletal dysmorphology of the long-face syndrome. Face symmetry and proportion, fundamental shape, along with traits like full cheeks, wide eyes, and a distinct nose, constitute a typical normal face. In a normal face, the upper third, middle third, and lower third of the face are apparently symmetrical. There was a significant correlation, i.e., positive, between anterior facial height, which was increased, and the vertical height of the maxilla [1]. VME is a condition where the upper jaw (maxilla) has excessive vertical growth in an inferior direction, leading to an imbalance in the face in the vertical dimensions [2]. Long faces, gummy smiles, and even open bites can result from this clinical condition. The causes of vertical maxillary excess can be multifactorial, including genetic factors, skeletal growth patterns, and environmental influences. The condition can have aesthetic and functional implications, affecting the appearance of the face and the proper functioning of the jaws. When someone has vertical maxillary excess, it can be challenging to shorten off a long face.

Treatment for vertical maxillary excess may involve orthodontic interventions, orthognathic surgery, or a combination of both, contingent upon the degree of the condition and the specific needs of the individual. In order to address a non-surgical correction of vertical maxillary excess, skeletal anchorage systems (SAS) have become increasingly popular [3-6].

The traditional understanding of anchorage management and biomechanics in orthodontics has been altered by temporary anchorage devices (TADs). They have simplified orthodontic treatment and replaced numerous conventional forms of mechanics [7-10]. One who first reported on the clinical use of TADs was 1983 Creekmore and Eklund [11] on a patient whose profound overbite was fixed with a Vitallium bone screw. The term "gummy smile" describes an excess of gingival tissue shown when smiling. Nowadays, gingival exposure of less than 2 mm is accepted as younger and aesthetically pleasing. A novel method of treating vertical maxillary excess is the skeletal anchorage system or SAS. SAS serves as an absolute anchorage control to rectify vertical maxillary excess in non-growing individuals in a short duration of time and is a minimally invasive procedure. Miniscrew implants (MSIs) or TADs, bone screws, and surgical mini plates form SAS [3,4,5,12-24].

VME presents a complex challenge in orthodontic treatment, necessitating effective anchorage systems for optimal correction. So, this research is useful to assess the skeletal anchorage system's (SAS) effectiveness in correcting the vertical maxillary excess among adult patients presenting with gummy smiles. 

## Review

Materials and methods

This research is a meta-analysis and systematic review, and this study concentrated on all research on clinical case reports that provided comprehensive clinical data on VME treated with SAS. Principles of justice, confidentiality, beneficence, and non-maleficence were all upheld throughout the investigation.

Research Question: “What is the effectiveness of the skeletal anchorage system as compared to the non-SAS treatment approach for correcting the vertical maxillary excess among adult patients presenting with gummy smiles?”

Protocol and Registration

The protocol for the study was created using the standard structured approach for systematic reviews (Preferred Reporting Items for Systematic Reviews and Meta-Analyses (PRISMA)), which is registered with Registration ID No. CRD42023493350 in the International Prospective Register of Systematic Reviews (PROSPERO).

By establishing two distinct reviewer teams, a classical team and a case, the overall protocol will set itself apart from previously established approaches. Reviewing the papers, including control groups, was the classical team. This group essentially carried out a systematic review, which was more conventional, excluding evidence from case series and studies/reports. A case report was evaluated by the case team. A comparative group was the case team, which served to find variations in the findings of the systematic review brought about by the use of data from case reports, case series, and studies. All of the teams have found papers that fit the predetermined inclusion criteria, carried out independent analyses and also risk of bias assessments, evaluated overall quality, and synthesized the strongest pieces of evidence. Throughout the entire procedure, the ultimate results of the opposite team were blind to either squad. Results from each team were presented, assessed, and contrasted once the systematic review study was finished [25]. In this research, no or zero patients were involved.

Eligibility Criteria

The present framework of the systematic review complies with patient, intervention, comparison, outcome and (sometimes) time (PICO(T)) criteria [26].

Study Designs

Control-group interventional studies (randomized controlled trials) and observational studies with and without groups that were exposed are excluded from this study [25].

Type of Population

Case reports with English full text, publications from 2008 to 2023, and analysis of the general global (18+) healthy adult human population with vertical maxillary excess or VME have been included. The case report, which fulfills predetermined standards that show they are ethical, scientifically sound, and well-documented under the CARE (for CAse REports) [27,28] guidelines and the Joanna Briggs Institute (JBI) Critical Appraisal Checklist for case studies/reports and for case series [29,30] that assign grades to case studies and reports based on data analysis, transparency, and completeness. Research that was carried out in an unethical manner, animal studies, abstract papers/conference proceedings, and opinion articles (or articles where full text is not available), letters to the editor, retrospective studies, language other than English, articles published in other databases, patients below 18 years, and non-human subjects (primates) were excluded. 

Type of Exposure/Intervention

The treatment of vertical maxillary excess with an absolute anchorage system, SAS, or TADs, was considered in this systematic review. Those studies were excluded in which correction of vertical maxillary excess with other treatment approaches was done.

Type of Comparators

We examined two review strategies in this protocol: one review methodology included high-quality case reports, whereas the other disregarded them. The comparator was the existence or lack of a stated, readily available control group that complies with ethical and scientific standards. If a control group was present, then it consisted of patients with vertical maxillary excess treated without SAS or no treatment [25-30].

Type of Outcomes

Main outcome(s): Clinical effectiveness of treatment of VME with the skeletal anchorage system was seen by reduction of vertical maxillary excess, intrusion of anterior teeth of maxilla, and improvement in the related cephalometric parameters. The cephalometric parameters considered in this study included both angular and linear measurements. The angular measurements were as follows: SN-PP; SN-GoGn; MP-MnOP; SN-MxOP; SN-MnOP; and SN-MP. The linear measurements included: OB; U1-PP; L6-GoGn; L1-GoGn; lower anterior face height (LAFH), and U6-PP.

Additional outcome(s): Improvement of the smile (i.e., gummy smile correction) and overbite.

Time

Case reports on SAS (or TADs) used for maxillary vertical excess correction in the English language published between 2008 and 2023 with full abstract accessibility were included.

Information Sources and Search Strategy

The search approach was a thorough search with no design restrictions. A thorough search was conducted using internet search engines, websites for organizations, electronic grey literature, and academic databases. We have searched the following significant academic electronic databases: Excerpta Medica database (EMBASE), Web of Sciences, Scopus, and PubMed.

In conducting this analysis, the PRISMA declaration [31] and PRISMA 2020 flow diagram for new systematic reviews, which only needed database and registration searches, were adhered to. Searches were restricted to a time frame of 2008 to 2023, and articles were considered only in English. The underlying search engines used for preliminary searches were carried out on December 6th, 2023, in all the above-mentioned databases. After that, on February 6th, 2024, using the same databases, the most recent search was carried out. The exact search is shown on the flow diagram of PRISMA 2020. We utilized a combination of major topics, indexed keywords, MeSH terms, and crafted our search strategy.

Boolean operators “AND” and “OR” were used for searching articles on PubMed [31-36]. Our search strategy central theme is mentioned in Table 1.

**Table 1:** Central theme of the study’s search strategy Search strategy for this study included the following data bases PubMed advanced search, Excerpta Medica database (EMBASE) (Elsevier), Web of Science, Scopus (advanced search)

S. No.	Database	Search strategy
1	PubMed advanced search	("gummy smile"[Title/Abstract] OR "vertical maxillary excess"[Title/Abstract] OR "VME"[Title/Abstract]) AND ("temporary anchorage device*"[Title/Abstract] OR "TAD"[Title/Abstract] OR "miniscrew"[Title/Abstract] OR "bone screw"[Title/Abstract] OR "SAS"[Title/Abstract] OR "skeletal anchorage system"[Title/Abstract]) (((((((("gummy smile") OR (vertical maxillary excess")) OR (VME)) AND (temporary anchorage devices)) OR (skeletal anchorage system)) OR (SAS)) OR (bone screw)) OR (miniscrews)) OR (TAD)
2	Excerpta Medica database (EMBASE) (Elsevier)	('gummy smile':ti,ab OR 'vertical maxillary excess':ti,ab OR VME:ti,ab) AND ('temporary anchorage device*':ti, ab OR TAD: ti, ab OR miniscrew: ti,ab OR 'bone screw':ti, ab OR SAS: ti,ab OR 'skeletal anchorage system':ti, ab)
3	Web of Science	("gummy smile" OR "vertical maxillary excess" OR VME) AND ("temporary anchorage device*" OR TAD OR miniscrew OR "bone screw" OR SAS OR "skeletal anchorage system")
4	Scopus (advanced search)	(TITLE-ABS("gummy smile") OR TITLE-ABS("vertical maxillary excess") OR TITLE-ABS(VME)) AND (TITLE-ABS("temporary anchorage device*") OR TITLE-ABS(TAD) OR TITLE-ABS(miniscrew) OR TITLE-ABS("bone screw") OR TITLE-ABS(SAS) OR TITLE-ABS("skeletal anchorage system"))

Selection Process

Every one of the two separate reviewer teams, the classical team and the case team, has submitted the search results for the literature to the Rayyan software (Rayyan Systems Inc., Cambridge, MA) separately and in compliance with the eligibility requirements after conducting a thorough search in each database. Duplicate entries were found and removed, and all the study records found throughout the search process were downloaded. The titles of potentially pertinent papers were then separately evaluated by two members of the study team, who then proceeded to screen the abstracts (step one) and complete texts (step two) for inclusion. In a few instances, the publication authors were asked for information to address eligibility concerns. Lastly, any potential conflicts between the two members of the study team were settled by first initial discussion and then followed by arbitration consultation with a third research team member. A third reviewer team did the final check of the included studies and the reasons and details of the inclusion or the exclusion of the studies.

Data Extraction

Two independent reviewer teams extracted data, and the third reviewer team did the final check of the included studies; the third reviewer team also resolved any disputes during the procedure of data extraction that could not be settled by agreement between the two original data extractor team members [25]. The data was extracted using MS Excel software (Microsoft Corporation, Redmond, Washington, United States). The primary outcome variables were taken into account during the data extraction process. The present study helps in extracting data in terms of population demographics (age, gender, area of study), participants, intervention/exposure, outcome, and study design. The study also includes the baseline characteristics of the orthodontic device placement, the anchorage system used, and details of the material used. The final outcome measures SAS or TAD usage and the success of reducing the vertical maxillary excess along with the improvement of the smile line [36-39].

Risk of Bias (Quality) Assessment

The National Institutes of Health (NIH) Quality Assessment Tool was employed to evaluate the articles that were ultimately chosen. There are fourteen questions on the NIH Quality Assessment Tool checklist. Every question pertinent to the included study designs that received a suitable response received a score of one; if not, it received a score of zero. In order to determine an individual score, questions that were not pertinent to the studies were excluded and did not count in the denominator. Here in this present study only those questions were considered which are suitable for this systematic review [40]. On the nhlbi.nih.gov website, the complete NIH tool can be found. 

Strategy for Data Synthesis

We included 12 studies that measured the progress of SAS or TADs in correcting maxillary excess.

Independently, the classical and case teams carried out data synthesis. For case series and case reports/studies, the initial proof grade was set at "low." It should be made clear that without study comparators, adequate levels of evidence cannot be obtained. Since case reports are categorized as beginning at the lowest level of evidence, we are unable to take evidence/proof greater than low into account for these kinds of research [25,40].

The Meta XL (v.5.0) program's (EpiGear International Pty Ltd., Queensland, Australia) Excel extension for meta-analysis was used to calculate the effectiveness of treating vertical maxillary excess among non-growing adult patients with skeletal anchorage systems. The inverse variance heterogeneity model was applied during the meta-analysis process [28,29]. I2 statistics and Cochrane's Q were applied to gauge the degree of data heterogeneity.

Statistical Analysis

Pooled intrusion and its confidence interval (CI) were the summary measures. We calculated the relative intrusion from those studies reporting only the intrusion with 95% CI. A public health dentist and biostatistician (DB) performed the meta-analysis if the studies were homogenous. Study authors were contacted when data was missing or inadequate. The data for all the outcomes were combined using the random effects DerSimonian Laird model. The pooled relative reduction was reported with 95% confidence intervals (CIs). To evaluate the heterogeneity among the individual studies, we reported the heterogeneity index (Q), degrees of freedom (df), and I2 statistics. An I2 value higher than 50% was deemed significant heterogeneity due to the intervention. The Meta Excel extension in MS Excel is used for both statistical analysis and graphical representation.

Results

**Figure 1:**
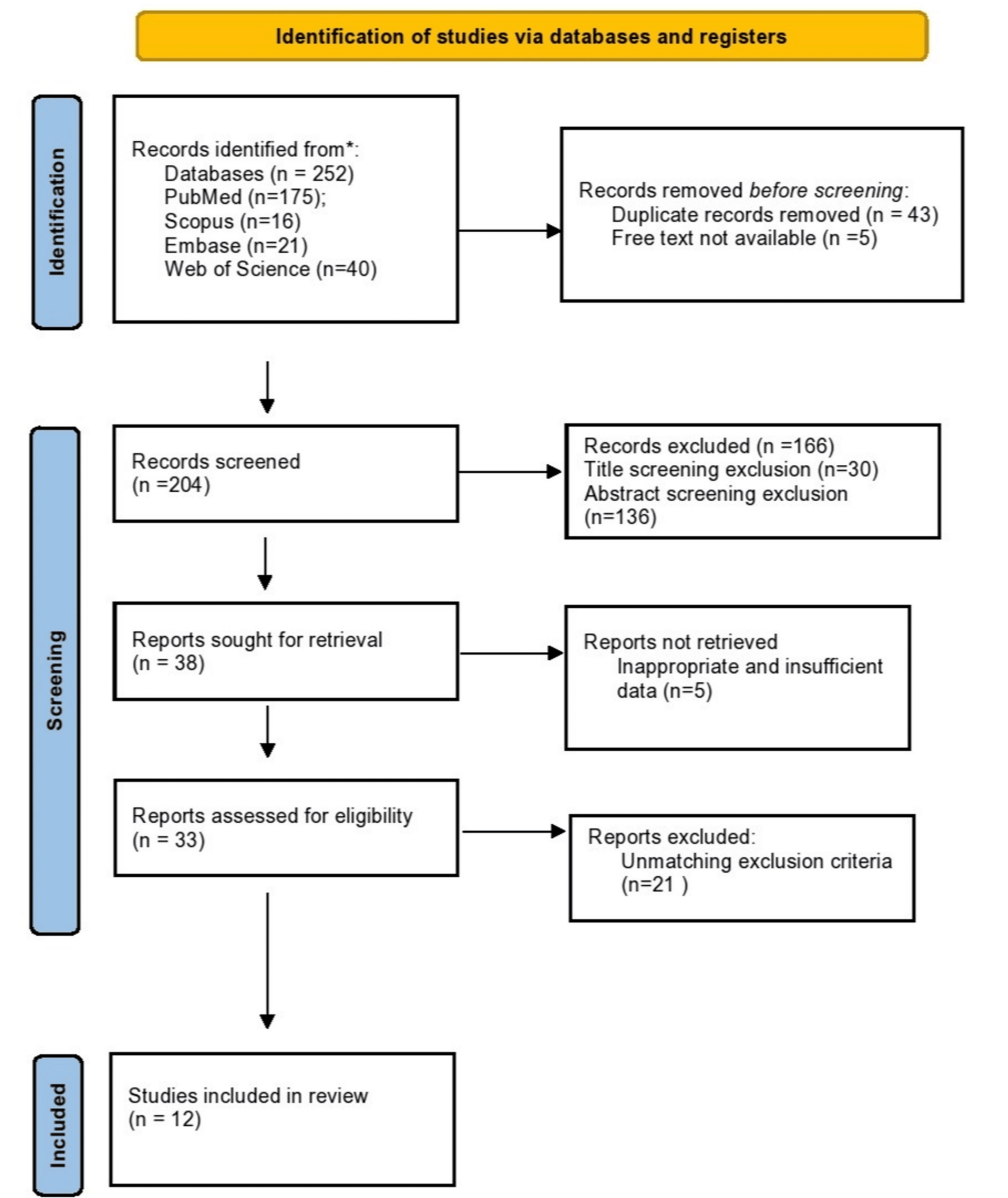
PRISMA 2020 flow diagram of the study according to the PRISMA guidelines (The PRISMA 2020 statement: an updated guideline for reporting systematic reviews) PRISMA: Preferred Reporting Items for Systematic Reviews and Meta-Analyses

Two hundred fifty-two potential articles were found due to the search technique; 175 were found on PubMed, 16 on Scopus, 40 on Web of Science, and 21 on Embase. At the start of the study, 43 duplicates were removed; five free texts were not available and were also excluded. Then, out of 166 records that were excluded, 30 were title screening exclusions and 136 were abstract screening exclusions. Following this, five records were not retrieved, and 21 papers were removed, such as studies of non-human origin/Fem, systematic/narrative review/meta-analysis, studies not related to this research eligibility/inclusion criterion, and commentaries. As a result, a total of 12 studies went through the process of analysis. The full process is explained in the PRISMA 2020 [31-36] flow diagram. Table 2 and Figure 1 (flow diagram) list the main components of each study that were taken into account.

**Table 2:** Summary of all the 12 studies included in this review The studies involved: Kaku M et al., 2012 [17], She T et al., 2017 [21], Hong RK et al., 2013 [19], Wong A et al., 2018 [22], Tanaka E et al., 2008 [15], Fawas M et al., 2022 [6], Lahoti E et al, 2018 [5], Shu R et al., 2011 [16], Paredes-Gallardo V et al., [23], Venugopal A et al., 2020 [24], Nishimura M et al., 2014 [20], Ishida Y et al., 2017 [4]

S. No.	Author Name (Year)	Title	Outcome
1	Kaku M et al., 2012 [17]	Gummy smile and facial profile correction using miniscrew anchorage	They were able to obtain a regular overjet and overbite. A Class I molar connection was attained, and the upper and lower dental arches were in good alignment. A 4-mm intrusion was also visible in the anterior teeth. Both the Z-angle and the ANB angle shifted from 56.5u to 70.0u and 6.6u to 5.8u, respectively.
2	She T et al., 2017 [21]	Interdisciplinary management of an orthodontic patient with temporomandibular disorder	The canine relationship was in Class I, the molar relationship was completed in full unit Class II, and the overjet altered from 4.5 mm at the centric relation to 2.5 mm during splint therapy. SNA decreased from 85° to 83°, while ANB decreased from 8° to 6°. 3 mm of intrusion.
3	Hong RK et al., 2013 [19]	Orthodontic treatment of gummy smile by maxillary total intrusion with a midpalatal absolute anchorage system	By the end of treatment, the patient's grin looked better with a harmonic occlusion. The overbite and overjet were overcorrected to an edge-to-edge bite, and a Class I occlusion was attained. 2.5 mm of intrusion.
4	Wong A et al., 2018 [22]	Conservative management of skeletal Class II malocclusion with gummy smile, deep bite, and a (palatally) impacted maxillary canine	Lip strain significantly improved. Optimal correction of the posterior buccal crossbite, gummy smile, deep bite, and palatally impacted canine. With a score of two points, the dental aesthetic outcome was outstanding. 2 mm of intrusion.
5	Tanaka E et al., 2008 [15]	Skeletal anchorage for orthodontic correction of severe maxillary protrusion after previous orthodontic treatment	Overall facial equilibrium was improved, according to facial photos. Upon closing, the lips displayed less strain. The overbite was adjusted to 1.2 mm and the overjet to 1.0 mm, and acceptable occlusion was attained. On both sides, the molar relationships were modified to Class I and an intrusion of 2.2 mm.
6	Fawas M et al., 2022 [6]	Gummy smile correction with miniscrews in Class II vertical maxillary excess	Decreased incisor visibility and interlabial space at repose. Class II molar on the right, Class I molar on the left, and Class I canine and incisor connection all show good occlusal relationships. The Wits value went from 2.5 mm to 2 mm, while the ANB was reduced by 2.50. The gap between the upper incisor and the lip line (U1-lip line) is 4 mm, 6 mm intrusion.
7	Lahoti E et al, 2018 [5]	Correction of gummy smile in a patient of vertical maxillary excess using absolute anchorage system	It has reached normal overjet and overbite. There was a 3 mm decrease in lower anterior face height. The chin and lips seemed more in tune. Intrusion of 4 mm.
8	Shu R et al., 2011 [16]	Adult Class II Division 1 patient with severe gummy smile treated with temporary anchorage devices	The lip profile was noticeably improved in the posttreatment photos. The posttreatment clinical assessment revealed no gummy smile. The mandibular first molars shifted 3.5 mm mesially, and the maxillary incisors' incisal edge retracted by 7 mm and intruded by 2.5 mm. The overjet and overbite were normal.
9	Paredes-Gallardo V et al., 2019 [23]	Miniscrew mechanics for molar distalization and incisor intrusion in a patient with a Class II brachyfacial pattern and gummy smile	Pictures of the face taken after treatments showed the attainment of a harmonic and balanced face. The wits appraisal went from 4.7 mm to 0.2 mm, the intrusion of 1.8 mm, and the ANB angle dropped from 5.6 to 4.6.
10	Venugopal A et al., 2020 [24]	Treating a severe iatrogenic gingival exposure and lip incompetence – a challenge worthwhile	A bilateral relation of Class I canine and molar, lip competence, and harmony between the upper and lower lips were also attained. This greatly enhanced the sagittal jaw connection. Both the lower and upper incisor inclinations significantly decreased. Ninety percent of the overjet was fixed, resulting in a final overbite of 1.5 mm. Intrusion of 4 mm.
11	Nishimura M et al., 2014 [20]	Non-extraction treatment with temporary skeletal anchorage devices to correct a Class II Division 2 malocclusion with excessive gingival display	From Class II to Class I, the canine and molar connections were normalized. The maxillary first molars were distalized by 4.0 mm and intruded by approximately 1.5 mm. The central incisors of the maxilla were 3.5 mm intruded. The mandible did not rotate as much counterclockwise as expected. The deep bite, gummy smile, and balanced profile were all drastically altered.
12	Ishida Y et al., 2017 [4]	Nonsurgical treatment of an adult with a skeletal Class II gummy smile using zygomatic temporary anchorage devices and improved superelastic nickel-titanium alloy wires	Both lips' protrusion and the tension on the mentalis following closure were removed, despite the fact that the height of the lower anterior face had barely changed. Normal overbite, overjet, and Class I molar relationship. There was a 3.4 mm intrusion and a 3.6 mm distalization of the maxillary first molars. At the incisal edge, the maxillary central incisors were distalized by 8.5 mm and intruded by roughly 2.2 mm. A beautiful grin that significantly improved the gummy smile.

Study Characteristics

The 12 studies (case reports) included a total of 12 participants from the global population, including India, the USA, Korea, Japan, China, and Mongolia, including 11 females and one male. All the studies are carried out between 2008 and 2022. The age of patients is between 18 and 36 years. Out of 12 studies, in 11 studies, the orthodontic problem was vertical maxillary excess in Class II malocclusion. In two studies, anchorage plates were used, and all the other ten studies used miniscrew anchorage with infrazygomatic crest implants (IZC) in many studies. The site of placement of SAS was in the posterior region of the maxilla in most of the studies. In a few studies, the anterior maxillary region was also used for TAD placement. In most studies, the duration of orthodontic treatment was two years. In four studies, the duration of treatment was more than two years. The post-treatment follow-up was for more than one year in most of the studies, except for two studies with less than one year of follow-up. The full study features are shown in Table 2, including study design [1-25].

Quality assessment of the included studies is given in Table 3. This table includes only those questions that are suitable for this systematic review [40].

**Table 3:** Quality assessment form of the included studies

Author name	Domains	Reporting of inclusion and exclusion criteria	Exposure adequately ascertained	Outcome adequately ascertained	Follow up long enough for outcomes	Reporting
1. Kaku M et al., 2012 [17]	Yes	Yes	Yes	Yes	Yes	Yes
2. She T et al., 2017 [21]	Yes	Yes	Yes	Yes	Partial	Yes
3. Hong RK et al., 2013 [19]	Yes	Yes	Yes	Yes	Yes	Yes
4. Wong A et al., 2018 [22]	Yes	Yes	Yes	Yes	Partial	Yes
5. Fawas M et al., 2022 [6]	Yes	Yes	Yes	Yes	Yes	Yes
6. Lahoti E et al, 2018 [5]	Yes	Yes	Yes	Yes	Partial	Yes
7. Shu R et al., 2011 [16]	Yes	Yes	Yes	Yes	Partial	Yes
8. Paredes-Gallardo V et al., [23]	Yes	Yes	Yes	Yes	Partial	Yes
9. Venugopal A et al., 2020 [24]	Yes	Yes	Yes	Yes	Yes	Yes
10. Nishimura M et al., 2014 [20]	Yes	Yes	Yes	Yes	Partial	Yes
11. Ishida Y et al., 2017 [4]	Yes	Yes	Yes	Yes	Partial	Yes

**Figure 2:**
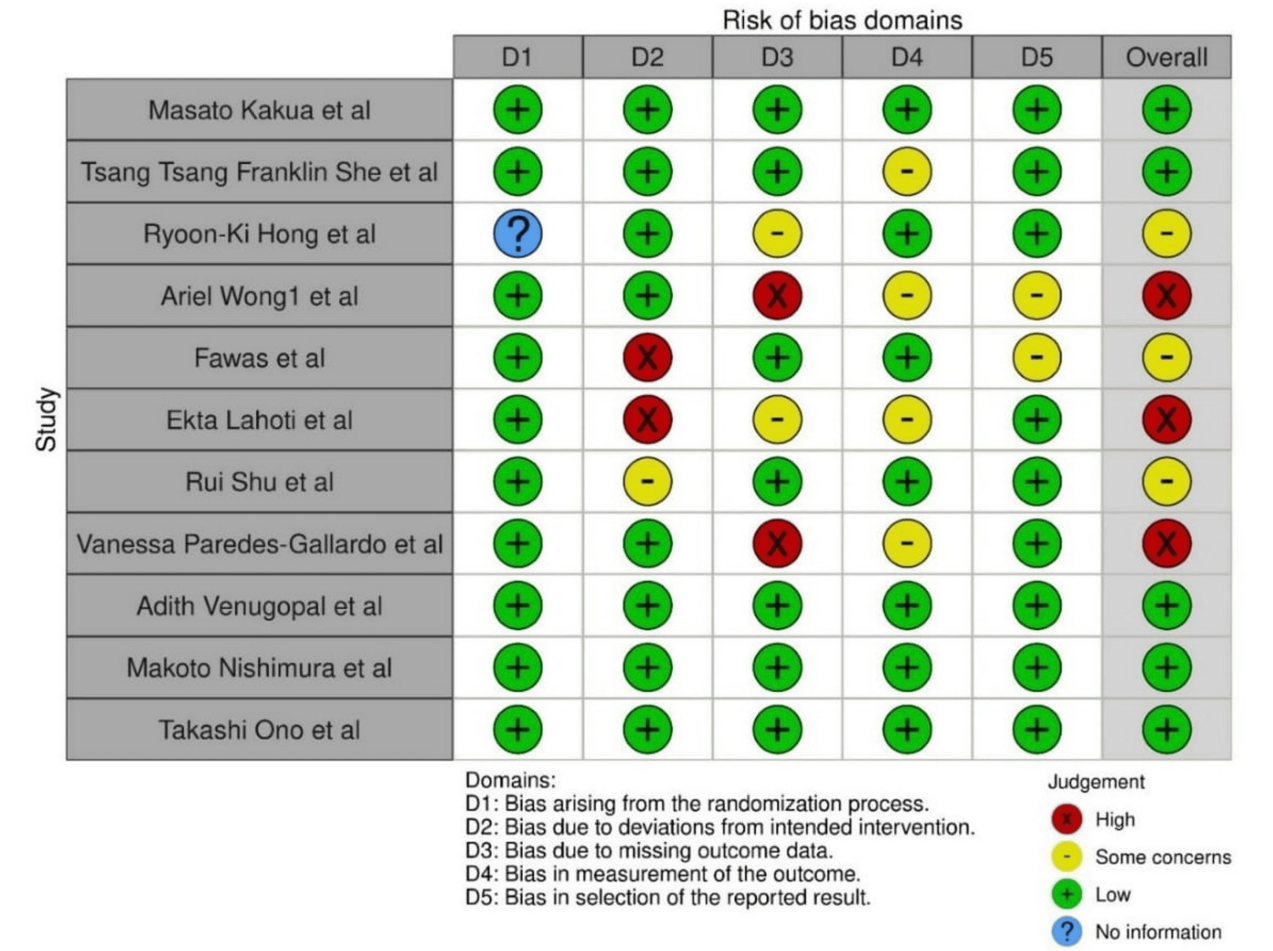
Risk of bias for individual studies The studies involved: Kaku M et al., 2012 [17], She T et al., 2017 [21], Hong RK et al., 2013 [19], Wong A et al., 2018 [22], Fawas M et al., 2022 [6], Lahoti E et al, 2018 [5], Shu R et al., 2011 [16], Paredes-Gallardo V et al., [23],  Venugopal A et al., 2020 [24], Nishimura M et al., 2014 [20], Ishida Y et al., 2017 [4]

**Figure 3:**
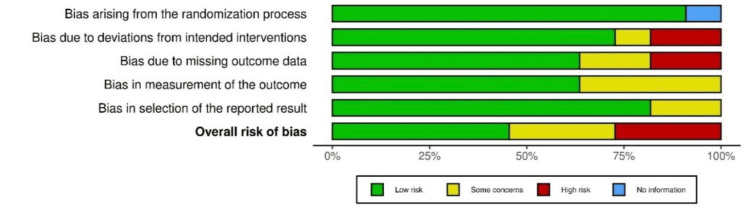
Pooled risk of bias for all the studies The studies involved: Kaku M et al., 2012 [17], She T et al., 2017 [21], Hong RK et al., 2013 [19], Wong A et al., 2018 [22], Fawas M et al., 2022 [6], Lahoti E et al, 2018 [5], Shu R et al., 2011 [16], Paredes-Gallardo V et al., [23],  Venugopal A et al., 2020 [24], Nishimura M et al., 2014 [20], Ishida Y et al., 2017 [4]

In Figure 2, for individual studies, the risk of bias is depicted, and for all the studies, the pooled risk of bias is depicted in Figure 3.

**Figure 4:**
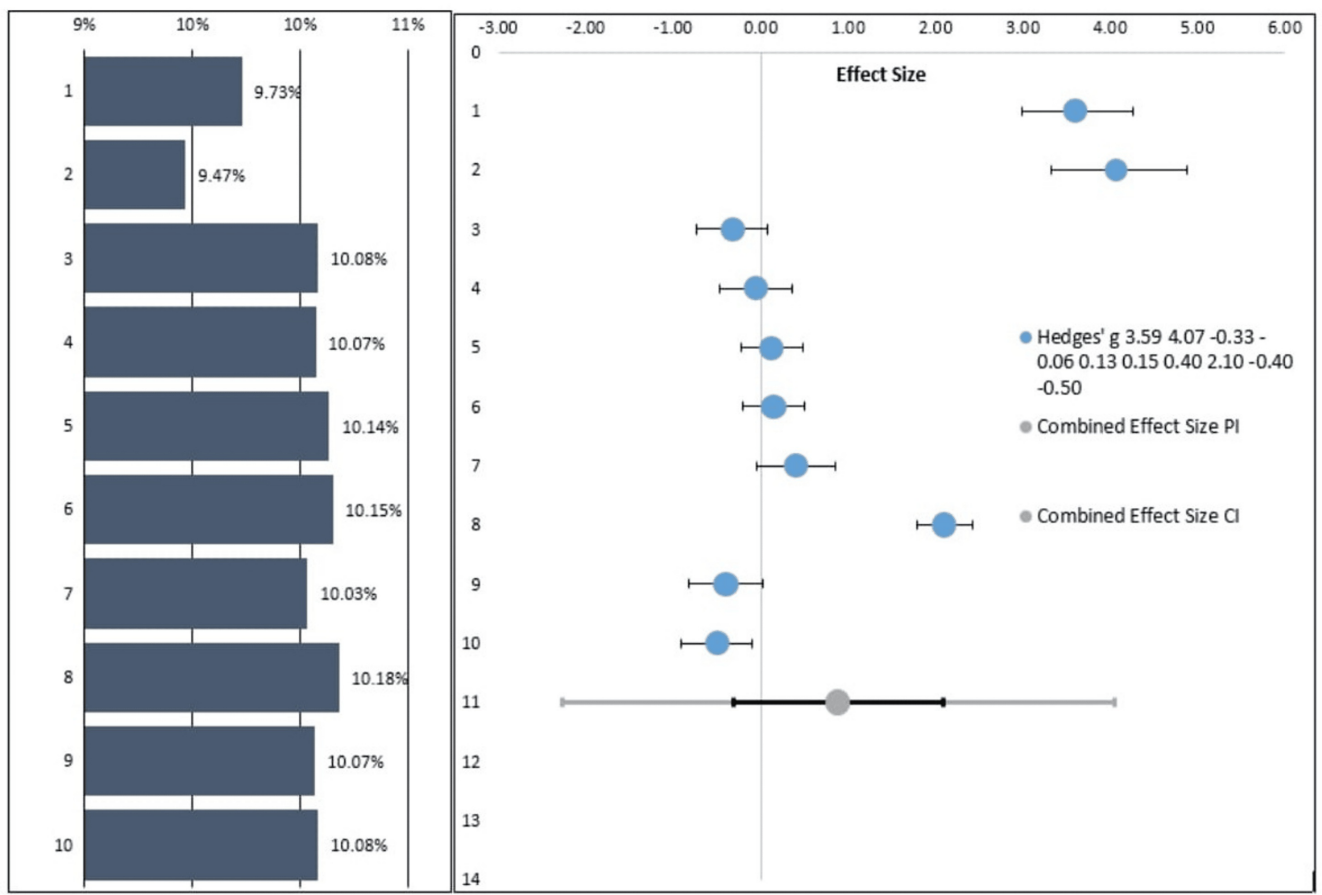
Forest plot showing the effect size of the improvements observed with the reports from different case reports The studies involved: Kaku M et al., 2012 [17], Hong RK et al., 2013 [19], Tanaka E et al., 2008 [15], Fawas M et al., 2022 [6], Lahoti E et al, 2018 [5], Shu R et al., 2011 [16], Paredes-Gallardo V et al., [23], Venugopal A et al., 2020 [24], Nishimura M et al., 2014 [20], Ishida Y et al., 2017 [4]

The details of the forest plot have been mentioned in Figure 4. It represents the study details and the effect size comparisons. In this particular case series, the pooled intrusion for the orthodontic treatment was reported, and it was seen that almost 70% of the studies had a similar range. Only three studies were out of the 95% CI range of the other studies. The p (p < 0.0001) was found to be statistically significant. CI intervals of the individual case reports have been mentioned in Table 4. The heterogeneity among the studies was 97.42% (Table 5).

**Table 4:** CI intervals of the individual case reports

Study name	Hedges' g	CI lower limit	CI upper limit	Weight
Kaku M et al., 2012 [17]	3.59	2.98	4.26	9.73%
Hong RK et al., 2013 [19]	4.07	3.33	4.89	9.47%
Tanaka E et al., 2008 [15]	-0.33	-0.74	0.08	10.08%
Fawas M et al., 2022 [6]	-0.06	-0.48	0.35	10.07%
Lahoti E et al, 2018 [5]	0.13	-0.23	0.49	10.14%
Shu R et al., 2011 [16]	0.15	-0.20	0.49	10.15%
Paredes-Gallardo V et al., [23]	0.40	-0.04	0.85	10.03%
Venugopal A et al., 2020 [24]	2.10	1.79	2.42	10.18%
Nishimura M et al., 2014 [20]	-0.40	-0.82	0.02	10.07%
Ishida Y et al., 2017 [4]	-0.50	-0.91	-0.10	10.08%

**Table 5:** Heterogeneity

Heterogeneity	
Q	348.21
p_Q_	0.000
I^2^	97.42%
T^2^	1.66
T	1.29

Publication Bias

**Figure 5:**
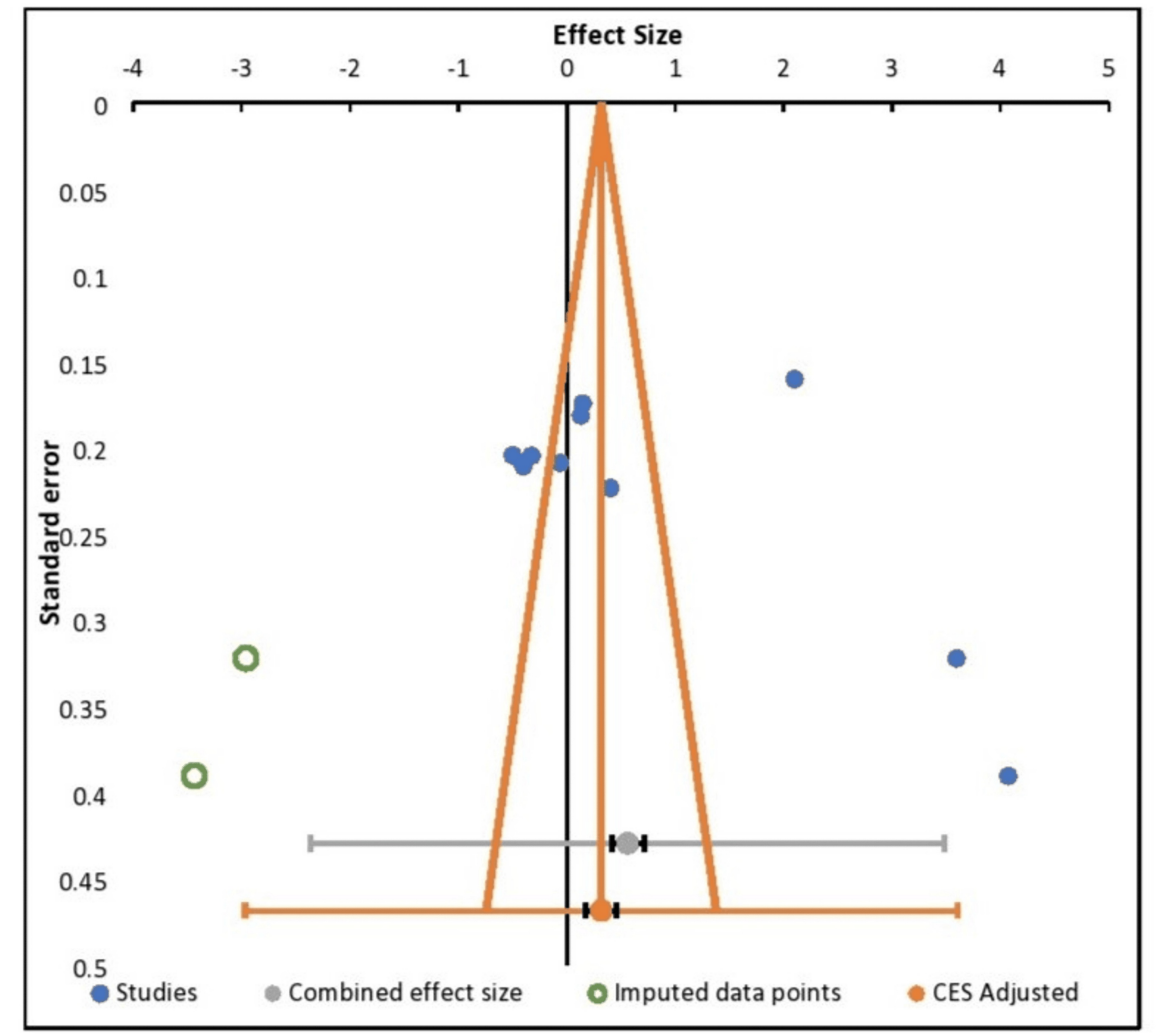
Funnel plot to represent the publication bias of the included case reports The studies involved: Kaku M et al., 2012 [17], Hong RK et al., 2013 [19], Tanaka E et al., 2008 [15], Fawas M et al., 2022 [6], Lahoti E et al, 2018 [5], Shu R et al., 2011 [16], Paredes-Gallardo V et al., [23], Venugopal A et al., 2020 [24], Nishimura M et al., 2014 [20], Ishida Y et al., 2017 [4]

A subjective analysis of the funnel plot revealed asymmetry that was mild; however, Egger’s regression test was performed, which elicited a p = 0.80, indicating the publication bias had minimal presence or was not very prevalent. Figure 5 represents the results from the funnel plot of case report studies regarding the effectiveness of treating vertical maxillary excess among non-growing adult patients with skeletal anchorage systems by the Egger test. There is an asymmetric inverted funnel shape, and the results suggest the possibility of publication bias.

Discussion

VME presents a complex challenge in orthodontic treatment, necessitating effective anchorage systems for optimal correction. A multitude of studies from various countries have investigated the efficacy of different anchorage systems in addressing VME [1,2]. Traditional anchorage methods that were used from historical times are conventional anchorage methods like headgear and intraoral elastics, which have been cornerstone approaches in orthodontic treatment. But this treatment approach is limited to growing patients. A study by Proffit et al. (2001) conducted in the United States evaluated the role of headgear in correcting VME, and their findings suggested that while headgear can provide some degree of anchorage control, it may not be sufficient to address severe cases of VME, necessitating adjunctive approaches [3,14]. Skeletal anchorage, or orthognathic surgery, was used to achieve optimal outcomes in patients with significant vertical discrepancies [1-3]. 

VME is addressed via using a traditional Le Fort I osteotomy. One of the several risks associated with Le Fort I osteotomy is with damage to the descending palatine artery (DPA). In an attempt to prevent issues pertaining to the Le Fort 1 osteotomy, a few changes have been made to the Le Fort 1 technique. These include Le Fort 1 with a horseshoe osteotomy, including a unilateral type, a U-shaped osteotomy, a pyramidal osteotomy, and a standard and modified horseshoe. In Japan, some research on these changes has been carried out [2,9,14].

Skeletal anchorage systems have revolutionized orthodontic treatment and have emerged as versatile tools for enhancing anchorage control in orthodontic treatment, offering precise control over tooth movement and anchorage reinforcement in correcting vertical maxillary excess [11]. Skeletal anchorage systems, including mini-implants and miniplates (also called temporary anchorage devices (TADs)), gained prominence globally as promising options for managing VME in non-growing patients. A systematic review by Papageorgiou et al. (2015), which included studies from Europe, Asia, and North America, concluded that skeletal anchorage systems significantly enhance anchorage control, allowing for more precise and predictable correction of VME compared to traditional methods [5,10].

Deema Alshammery et al. (2021) [18] conducted a systematic review repairing gummy smiles non-surgically utilizing temporary skeletal mini-screw anchorage devices and discovered that TSAD are a useful and practical way to help reduce excessive gingival show or gummy smile.

Yuji Ishida et al. (2017) found suitable dental occlusion, aesthetic enhancement, and acceptable function after maxillary dentition intrusion and distalization with skeletal anchorage. They added that this treatment method, which is for adults with a gummy smile and skeletal Class II malocclusion with high-angle, improved with zygomatic temporary anchorage devices and superelastic nickel-titanium alloy wires, ought to be taken into consideration as a substitute for orthognathic surgery [4]. Similar findings were also observed by Ekta Lahoti et al. (2018) [5] and Adith Venugopal et al. (2020) [24].

She et al. (2017) observed whole arch intrusion as well as the intrusion of upper dentition and reduced overjet; they also observed more bone volume for uprighting upper incisors, and there was no posterior interference. Gummy smile correction was achieved with miniscrew anchorage [21]. Wong A et al. (2018) found correction of the impacted (palatally) canine; they also observed correction of buccal crossbite in the posterior region, deep bite correction, strain in the lip, and gummy smile correction using anchorage provided by maxillary anterior miniscrews and infrazygomatic crest bone screws [22]. 

Vanessa Paredes-Gallardo et al. (2019) found 1.8 mm for maxillary and mandibular incisors and 2.1 mm of intrusion, respectively. They employed miniscrew mechanics for incisor intrusion and molar distalization in a brachyfacial pattern patient with malocclusion, i.e., Class II and gummy grin. The occlusion and facial aesthetics attained at the conclusion of treatment were flawlessly preserved during a 24-month retention period [23].

Mohammed Fawas et al. (2022) found that after first premolar extraction, dual buccal miniscrews are a great way to treat VME in hyperdivergent Class II patients. This can result in maxillary arch intrusion, which corrects gummy smiles, autorotation of the mandible, and LAFH decrease, all of which enhance the facial profile. Thus, orthodontic treatment, as opposed to orthognathic treatment, can be used for adult patients with gummy smiles [6].

Masato Kaku et al., Rui Shu et al., Makoto Nishimura et al., Yuji Ishida et al., and Adith Venugopal et al. reported adequate intrusion of the upper anterior. Makoto Nishimura et al., Tsang Tsang Franklin She et al., and Mohammed Fawas et al. (2022) reported maxillary total intrusion, which they supported with evidence of cephalometric parameters. They also found that there was autorotation of the mandible in an anticlockwise direction and LAFH reduction, which thus improves the profile [4,6,16,17,20,21,24].

The relevant body of literature already contains all of the research that are part of this systematic review, and these studies suggest many relevant cephalometric parameter changes that were achieved by the SAS and are useful as well as helpful in improving the overall patient profile [1-24,41-58].

In this particular case series, the pooled intrusion for the orthodontic treatment was reported, and it was seen that almost 70% of the studies had a similar range. Only three studies were out of the 95% CI range of the other studies. The heterogeneity among the studies was 97.42%. It was discovered that the p was statistically significant. (p < 0.0001). On a forest plot, each horizontal line denotes a single study with the result represented as a box, and the line displays the 95% confidence interval. Each study falling on one side of the vertical line or the other depends on the statistic being used.

It was seen that the study conducted by Kaku M et al., 2012 [17] had an effect size of 3.59; Hong RK et al., 2013 [19] had an effect size of 4.07; Tanaka E et al., 2008 [15] had an effect size of -0.33; Fawas M et al., 2022 [6] had an effect size of -0.06; Lahoti E et al., 2018 [5] had an effect size of 0.13. Shu R et al., 2011 [16] had an effect size of 0.15; Paredes-Gallardo V et al. [23] had an effect size of 0.40; and Venugopal A et al., 2020 [24] had an effect size of 2.10; Nishimura M et al., 2014 [20] had an effect size of -4.0; and Ishida Y et al., 2017 [4] of 0.50. The pooled estimate was around 1. Cohen (1988, 1992) offered criteria for interpreting these values: Large, medium, and minor effects are generally seen to be represented by Hedges' g values of 0.80, 0.50, and 0.20, respectively.

Limitations

This study is a systematic review and meta-analysis of case reports.

Recommendation and Future Scope

Correction of VME and gummy smile was achieved with SAS, and also there was post-treatment stability after two years. So, we suggest the usage of SAS to achieve correction of patients with mild to moderate vertical maxillary excess. In the future, more research works are required to enlighten the efficiency and efficacy of all SAS for the correction of VME and other dentoskeletal problems.

## Conclusions

Skeletal anchorage systems play a pivotal role in improving vertical maxillary excess among orthodontic patients across diverse populations. Skeletal anchorage systems offer effective solutions for enhancing anchorage control and achieving optimal correction of mild to moderate VME cases without the need for orthognathic surgery (OGS), thus providing a viable alternative to the patients. The combination of gingivectomy and intrusion provides an aesthetically attractive profile and smile without the expense, risk, and any potential side effects of OGS. Moreover, multidisciplinary collaboration is essential for the comprehensive management of severe VME cases, thus integrating orthodontic treatment with surgical interventions when necessary. Further research and clinical studies from various countries are warranted to refine anchorage techniques and optimize treatment outcomes for patients with VME.
